# Involving Patients in Weighting Benefits and Harms of Treatment in Parkinson's Disease

**DOI:** 10.1371/journal.pone.0160771

**Published:** 2016-08-30

**Authors:** Marieke G. M. Weernink, Janine A. van Til, Jeroen P. P. van Vugt, Kris L. L. Movig, Catharina G. M. Groothuis-Oudshoorn, Maarten J. IJzerman

**Affiliations:** 1 Department of Health Technology and Services Research, MIRA institute for Biomedical Technology and Technical Medicine, University of Twente, Enschede, the Netherlands; 2 Department of Neurology, Medical Spectrum Twente, Enschede, the Netherlands; 3 Department of Clinical Pharmacy, Medical Spectrum Twente, Enschede, the Netherlands; University of California Los Angeles, UNITED STATES

## Abstract

**Introduction:**

Little is known about how patients weigh benefits and harms of available treatments for Parkinson’s Disease (oral medication, deep brain stimulation, infusion therapy). In this study we have (1) elicited patient preferences for benefits, side effects and process characteristics of treatments and (2) measured patients’ preferred and perceived involvement in decision-making about treatment.

**Methods:**

Preferences were elicited using a best-worst scaling case 2 experiment. Attributes were selected based on 18 patient-interviews: treatment modality, tremor, slowness of movement, posture and balance problems, drowsiness, dizziness, and dyskinesia. Subsequently, a questionnaire was distributed in which patients were asked to indicate the most and least desirable attribute in nine possible treatment scenarios. Conditional logistic analysis and latent class analysis were used to estimate preference weights and identify subgroups. Patients also indicated their preferred and perceived degree of involvement in treatment decision-making (ranging from active to collaborative to passive).

**Results:**

Two preference patterns were found in the patient sample (N = 192). One class of patients focused largely on optimising the process of care, while the other class focused more on controlling motor-symptoms. Patients who had experienced advanced treatments, had a shorter disease duration, or were still employed were more likely to belong to the latter class. For both classes, the benefits of treatment were more influential than the described side effects. Furthermore, many patients (45%) preferred to take the lead in treatment decisions, however 10.8% perceived a more passive or collaborative role instead.

**Discussion:**

Patients weighted the benefits and side effects of treatment differently, indicating there is no “one-size-fits-all” approach to choosing treatments. Moreover, many patients preferred an active role in decision-making about treatment. Both results stress the need for physicians to know what is important to patients and to share treatment decisions to ensure that patients receive the treatment that aligns with their preferences.

## Introduction

Parkinson’s disease (PD) is a progressive and degenerative disorder which causes tremors and difficulties with walking, movement, and coordination. Treatment is aimed at maintaining continuous relief of motor symptoms and improving the patient’s functional capacity [1]. However, a significant number of patients with PD are still undertreated, e.g. because of suboptimal adherence to drug regimens, "levodopa phobia", or fear of deep brain stimulation [2, 3]. For example, Leopold et al. (2004) have shown that only 10% of patients with PD fully adhere to drug regimens as intended [4]. Besides treatment effectiveness, other attributes such as ease of use, routines, and treatment modality might influence patients’ decisions about treatment adherence or advanced treatments (subcutaneous or intraduodenal pump infusion or neurosurgery) [5–9].

Physicians have already begun to pay increased attention to patient-reported outcomes and started to recognise quality of life as the primary treatment goal [10, 11]. However, to further optimise treatment outcomes–especially outcomes that matter to the patient–physicians need to better understand how patients weigh up the benefits, side effects and process characteristics of treatment. Although it is a considerable challenge to find ways to elicit patients' preferences, the explicit weighing of treatment characteristics is essential to gather information about the relative desirability of treatment outcomes and modalities from the patient’s point of view [12]. Since it is often not possible to reduce all symptoms and side effects for a patient with PD, it is important to know whether a patient will, for example, accept the risk of suffering from incidental bouts of dizziness in order to obtain improved motor function.

Patients may weigh these benefits and harms differently to physicians which could influence the treatment patients end up with, depending on who is leading the choice of treatment [13]. Patient involvement is essential in deciding which treatment is best tailored to the individual patient's needs [14–16]. Moreover, patients are also more likely to be compliant and follow a treatment regimen if they experience greater involvement in the decision-making process [17].

Although patient-centred care is considered important in PD care, little is known about the degree of patient involvement in treatment decisions and the congruence with patients’ preferred involvement. Moreover, little is known about the actual trade-offs that patients would prefer to make between the benefits and side effects of treatments. In this study, we aim to elicit patient preferences around motor symptoms, side effects, and factors related to the delivery of care in the main treatments in PD (oral intake of medication, continuous pump infusion of medication, and neurosurgery). A second objective was to assess the congruence between patients’ preferred and perceived involvement in decision-making about treatment.

## Methods

In this study, the best-worst scaling case 2 method was used to elicit patient preferences about the relative desirability of treatment outcomes [18, 19]. In best-worst scaling respondents are presented with possible treatment scenarios, described according to their characteristics, and patients have to choose the best and worst features of possible treatments. In the next paragraph the way in which these treatment scenarios were created and presented will be explained.

### Best-worst scaling: creating treatment scenarios

In a best-worst scaling experiment, treatment scenarios are described by an underlying, basic set of characteristics of care (called attributes) and each attribute is represented by two or more values (called levels). In this study, 18 interviews with patients (qualitative research) provided the basis for identifying the full set of attributes that characterise PD treatments and influence patients’ Health Related Quality of Life (HRQoL). The results of this qualitative research are summarised below, but a more extensive report can be found in S1 Appendix.

Patients for this stage were recruited from the hospital Medisch Spectrum Twente (all 18 patients took oral medication, three patients have had neurosurgery in the past, and one patient received medication via a pump). Patients were first asked to describe their health using the Parkinson-Disease Questionnaire-39 (PDQ-39) [20] and the EuroQol-5D-5L (EQ5D-5L) [21]. The interviewer used this information to structure the interviews and to decide which health domains were important to discuss (semi-structured interviews). Next, patients were questioned about the impact of symptoms, side effects, and process characteristics on their HRQoL. The interviews were tape recorded and transcribed verbatim and coded and analysed in ATLAS.ti [22]. The final result was a table ranking the frequency of symptoms and side effects (attributes) and their impact on HRQoL according to these patients.

Yet best-worst scaling is typically limited to a small number of attributes (four to eight). Discussion with a project team consisting of a neurologist, a hospital pharmacist, a rehabilitation specialist, three health sciences researchers, and two patients was used to narrow the list of attributes. The task of the project team was to select a balanced set of symptoms, side effects, and process characteristics based on the qualitative and quantitative importance of these attributes of treatment in patients’ daily life (interview results), and to make sure that the selected attributes were typical symptoms and side effects of PD and not too general.

After deliberation, one process attribute (treatment modality), three motor symptoms (tremor, slowness of movement, and posture and balance problems) and three side effects (dizziness, drowsiness, dyskinesia) were selected for inclusion in the preference task. The selection of the process attribute reflected interviewed patients expressing concerns regarding the impact of surgery and pump procedures on their daily life, despite the expected reduction in symptoms. This highlights an important trade-off in expressing preference for treatment. For motor symptoms, the project team followed the interview results: tremor, slowness of movement, and posture and balance problems were the problems most frequently reported and all had a major impact on patient’s HRQoL. Less common symptoms were: problems with writing, crying, drooling, swallowing, loss of smell, and constipation. To keep a balance between the negative and positive effects of treatments, three side effects were also selected. The interview results showed that dyskinesia occurred the most often and had the largest impact on patients’ daily life, because of the duration, the unpredictable character, and the obstruction of daily tasks. Secondly, side effects were most often reported in the sleeping domain and the selected attribute ‘drowsiness’ was defined as extensive daytime sleepiness. Nausea, stomach pain, vomiting, diarrhoea, and constipation also occurred frequently, but the project team concluded that these side effects were too general. Lastly, dizziness (lightheadedness caused by orthostatic hypotension) was selected instead of ‘hallucinations and paranoia’. Although the latter had a larger impact on HRQoL, it was only reported by three patients.

**Fig 1:**
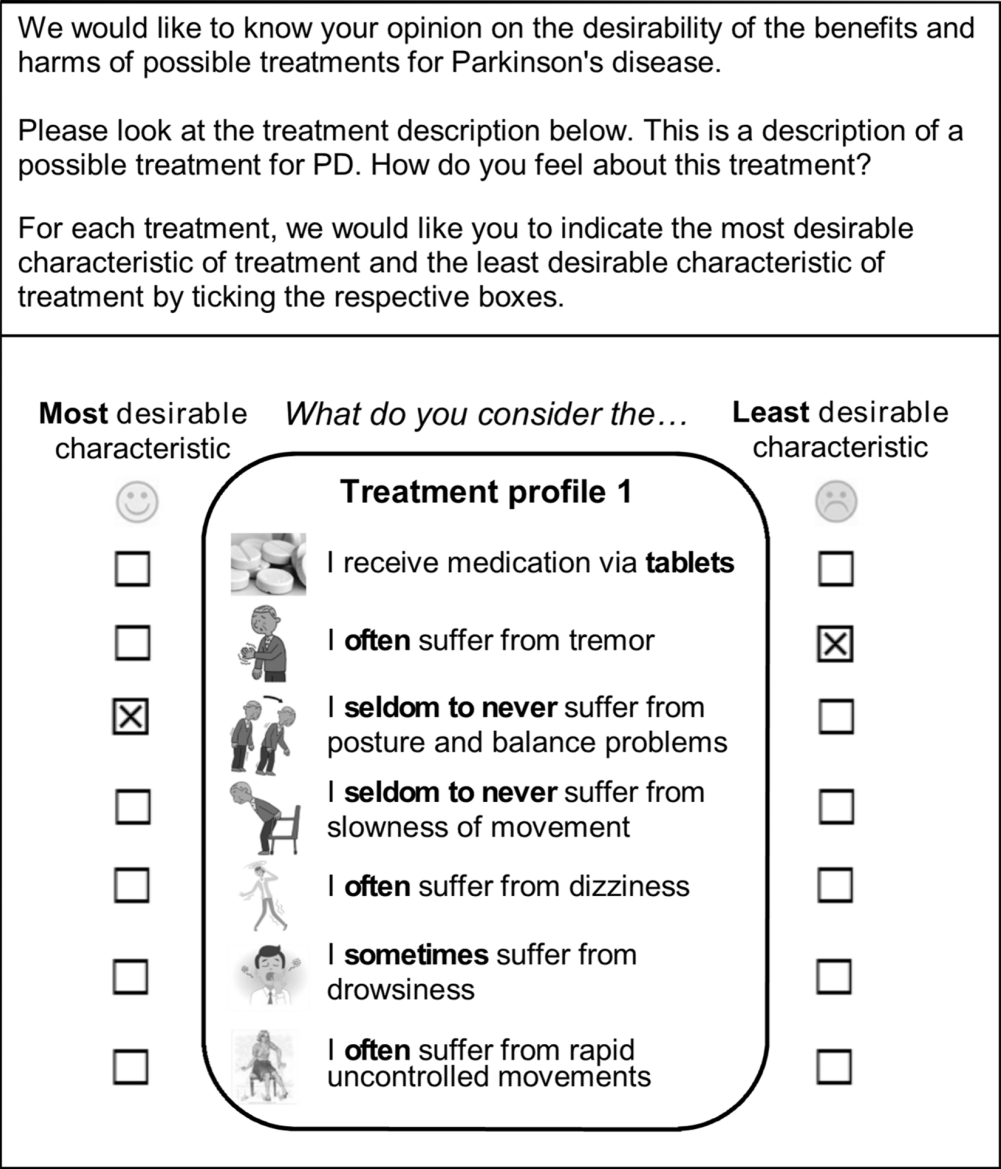
Example of a treatment profile in which the patient had to indicate the least and most desirable characteristic of treatment.

The next step was to describe the variation in possible outcomes for each attribute. In order to reduce the cognitive difficulty, three qualitative levels were chosen to represent the burden of symptoms and side effects in the treatment scenarios (seldom to never, sometimes, and often suffer from). The attribute treatment modality was described as the oral intake of tablets, continuous pump infusion of medication, and neurosurgery. The attribute-levels were systematically combined into treatment profiles and in theory 2187 (3^7^) hypothetical treatment scenarios were possible. Since it is impossible to ask patients to answer best-worst questions for each of these scenarios, experimental design software was used to select the smallest subset that identified all necessary parameters [23]. Experimental design software uses algorithms to construct D-optimal designs to approximate, so that each attribute-level appears the same number of times and each attribute-level appears an equal number times with another attribute-level (no correlation). Our final subset consisted of 36 profiles, which we divided over four versions of the questionnaire (nine per version). For each hypothetical treatment profile, patients were asked to indicate which aspect of treatment they found most and least desirable (Fig 1). Thus patients were asked to make a trade-off between treatment modality, symptoms, and side effects of treatments. By offering several of these scenarios to multiple patients, the data can be used to predict the relative desirability of treatment characteristics from the patients’ point of view (as a group). The best-worst scaling questions were introduced by a detailed explanation and a clear example. Respondents were provided with information sheets about the advanced treatments.

### Control Preference Scale

In the second part of the questionnaire, patients were asked about their perceived and preferred involvement in the choice of treatment. A modified version of the validated Control Preferences Scale was used to determine the level of agreement between the preferred and perceived decision role of the patient [24, 25] (Table 1). Since preferred and perceived decision roles were operationalised differently, the measurement of agreement also included patients who experienced only a slight difference in decision roles (e.g. patients who said they preferred an active shared decision role were included if they picked either the active or collaborative decision role as their perceived decision role). In addition, it was decided to only measure extreme discordance between decision roles. The validated Control Preferences Scale does not study the role of the caregiver or partner. Therefore, we questioned (in addition to benefits and harms) to what extent advice from family and friends and burden of treatment for partner/caregiver influenced decision-making (5-point rating scale: no influence to very influential). Lastly, patients were asked for socio-demographics, quality of life (PDQ-8 [26] and EQ5D-5L [21]), PD-related medication, and current/previous treatments.

**Table 1:** Questions and answer-categories used to assess patient’s preferred and perceived decision role in treatment decision making.

*Preferred Decision Role*: *To what extent do you want to be involved in the treatment decision?*	*Perceived Decision Role*: *To what extent were you involved in the previously made treatment decision?*
• I prefer to make the final treatment decision	• Treatment was largely chosen by myself
• I prefer to make the final treatment decision after seriously considering my doctor’s opinion	• Treatment was chosen in collaboration with the doctor
• I prefer that my doctor and I share responsibility for deciding which treatment is best	• Treatment was largely chosen by the doctor
• I prefer that my doctor makes the final treatment decision, but seriously considers my opinion	
• I prefer to leave all treatment decisions to my doctor	

### Study Population

A paper version of the questionnaire was sent to all patients diagnosed with idiopathic PD at the outpatient department of Medisch Spectrum Twente (except for patients who were registered as having atypical parkinsonism or dementia). Medisch Spectrum Twente is a large teaching hospital in the Netherlands that provides all three advanced therapies in PD (deep brain stimulation, subcutaneous apomorphine infusion and intraduodenal levodopa infusion). The data collection was expanded to include a non-hospital based patient sample by recruiting respondents through online Parkinson communities, the website and paper magazine of the Dutch Parkinson’s Disease Society and PD forums in both the Netherlands and Belgium. It could not be verified whether these participants were genuinely diagnosed with idiopathic PD or had atypical parkinsonism or dementia, because there was no access to hospital records. These patients were invited to complete the online survey or to request the paper version. According to the Medical-Ethics Twente Committee, this study did not have to be assessed against the medical research involving human subjects legislation. According to Dutch law, written informed consent was not required; by returning the completed questionnaire patients agreed to the use of anonymised data for the research purposes of this study.

### Data analysis

First, patients with incomplete data on both main parts (Control Preference Scale and best-worst scaling questions) were excluded. Subsequently, the data from the hospital and non-hospital patient samples were merged and socio-demographic, health outcomes, and treatment characteristics were described using frequencies, percentages, means, and standard deviations (SD).

For the best-worst scaling analysis, patients who had multiple response errors in their paper-questionnaire (ticked multiple best/worst boxes in one scenario, chose only the most desirable characteristics, or left multiple questions blank) were excluded. The online submitted surveys contained no missing data nor response errors, because error messages were presented during completion. Next, a conditional logit model estimated part-worth utilities for each attribute-level. Due to a latent scale, part-worth utilities cannot be directly compared. Importance weights were therefore calculated, based on the difference between minimum and maximum part-worth utilities within an attribute. The largest difference value received an importance weight of one, representing the attribute which had the highest impact on treatment desirability and the other difference values were divided by the largest difference value, resulting in a relative distance of all attributes to the attribute with the highest impact. Latent Class analysis was conducted to explore heterogeneity in preferences. Latent class analysis sorts patients into classes based on similarity in preferences and then identifies patient characteristics that are significantly associated with class membership. The model provides information about the likelihood of patients falling into as specific class on the basis of their characteristics [27]. The number of classes was pre-specified by fit indices (Bayesian and Akaike Information Criterion BIC/AIC) which measure the quality of statistical models. In this study, the AIC indicated a three-class model while the BIC indicated a two-class model. A two-class model was selected because using three classes did not reveal an additional interpretable subgroup. To determine whether certain patients were more likely to belong to one of the classes, several potential predictors were entered and tested for their significance (socio-demographics, health outcomes, experience with symptoms/side effects, treatment characteristics and quality of life measures). Finally, for the Control Preference Scale questions a chi-square test was used to evaluate the level of agreement between the perceived and preferred decision role of patients in decision-making about treatment (2-sided, p-value < 0.05). All data was analysed using Stata version 14 (StataCorp, College Station, TX).

## Results

132 patients from the Medisch Spectrum Twente sample returned their paper-questionnaire (response rate: 48%). Eight questionnaires were returned with incomplete data on all main parts of the questionnaire and were not used for further analysis. The non-hospital based patient sample consisted of 105 complete surveys online, with no paper versions requested. Most questionnaires (80%) were completed by patients independently, only 20% of patients were assisted by their partner or caregiver.

### Patient sample

Background characteristics and health care data for the total patient sample (N = 229) are summarised in Table 2. The sample consisted predominantly of men (66.5%), with a mean age of 65.4 years (SD 10.0) and a mean disease duration of 7.6 years (SD 6.9). Only 15% of patients were still employed. Concerning the clinical data, most patients faced mild to moderate impairments in motor symptoms: tremors (59.6%), slowness of movement (85.0%), and posture and balance problems (78.8%). About 81.3% sometimes or often suffered from drowsiness, 46.2% from dizziness (orthostatic hypotension), and 44.4% from dyskinesia. Most patients indicated moderate impairments in quality of life (PDQ-8: 31.0 (16.4), EQ5D-5L: 0.69 (0.17)), but 73% of patients were still able to live independently or only required help from their partner. However, almost three-quarters of patients reported one or more co-morbidities. Almost all patients took oral medication (96.1%), and advanced treatments were seen in only 17.5% of patients, with about 13.5% having had neurosurgery in the past and 4% currently wearing a pump. Apart from the non-hospital based sample being significantly younger and more often employed, both patient samples were comparable and they are therefore merged in Table 2.

**Table 2:** Background, socio-demographic and clinical characteristics (N = 229).

Variables	N (%) *or* mean ± SD (min, max)
**Background Characteristics & Socio-Demographics**	
**Sample**	
Medisch Spectrum Twente (hospital based sample)	124 (54.1)
Non-hospital based sample	105 (45.9)
**Gender**	
Man	151 (66.5)
Woman	76 (33.5)
**Marital state**	
Single	31 (13.7)
With partner (no children living at home)	152 (81.0)
With partner and children living at home	9 (4.0)
Other	34 (15.0)
**Age (years)**	65.4 ± 10.0 (33, 88)
Education level*	
Low	84 (37.0)
Medium	65 (28.6)
High	78 (34.4)
**Employment status**	
Employed (full or part-time)	34 (14.8)
Disabled / unable to work	52 (22.7)
Retired	135 (59.0)
House maker or housewife	8 (3.5)
**Questionnaire completed by**	
Patient	182 (80.5)
Patient and partner	33 (14.6)
Patient and caregiver (other than partner)	11 (4.8)
**Health Outcomes and Treatment Characteristics**	
**Disease duration (years)**	7.6 ± 6.9 (0.25, 31)
**Current treatment**^¥^	
Oral medication	220 (96.1)
Levodopa monotherapy	90 (39.3)
Dopamine agonist monotherapy	23 (10.0)
Combined levodopa/dopamine agonist treatment	107 (46.7)
Continuous pump infusion (subcutaneous)	2 (0.9)
Continuous pump infusion (intraduodenal)	7 (3.1)
Neurosurgery (one-sided)	7 (3.1)
Neurosurgery (two-sided)	24 (10.6)
**General health experience**	
(Very) good	69 (30.2)
Moderate	135 (59.2)
(Very) poor	27 (10.6)
**Severity of motor symptoms**	
No visible symptoms of PD	30 (13.3)
Symptoms are one-sided	113 (50.0)
Symptoms are two-sided	83 (36.7)
**Suffering from motor symptoms**	
Tremor	136 (59.6)
Posture and balance problems	180 (78.8)
Slowness of movement	195 (85.0)
**Suffering from side effects**	
Dizziness	106 (46.2)
Drowsiness	186 (81.3)
Dyskinesia	101 (44.4)
**Independency**	
Independent in self-care tasks	70 (30.7)
Assistance from family member (partner)	96 (42.1)
Home health care attendant	53 (23.2)
Living in assisted living facility	7 (3.1)
Other	2 (0.9)
**Patients with co-morbidities**^¥^	143 (74.5)
Hypertension	38 (19.8)
Heart disease	36 (18.8)
Sleep disorder	36 (18.8)
Arthritis	24 (12.5)
Cancer	10 (5.2)
Diabetes	7 (3.6)
Other	41 (21.4)
**PDQ-8**	
Felt depressed**	15 (6.7)
Had concentration problems**	45 (19.8)
Was unable to communicate properly**	36 (15.8)
**Quality of Life Measures**	
**PDQ-8 Summary Index Score (0–100)**	31.0 ± 16.4 (0, 71.9)
**EQ5D-5L Index Value (0–1)**	0.69 ± 0.17 (0.1, 1)
**Rating scale current health (0–100)**	64.9 ± 17.6 (1.0, 100)

* Low educational level: lower technical and vocational training and lower general secondary education; Medium education level: intermediate vocational training and advanced secondary education; High education level: higher vocational education and university.

** This was a single item in the PDQ-8. Here the patients are reported who sometimes, often or always experienced this item during the last month.

^¥^ Multiple replies possible.

### Treatment desirability in Parkinson’s Disease

Due to incomplete data or incoherent responses in the best-worst scaling questions, an additional 37 patients were excluded from this analysis. These excluded patients were significantly older than 75 and were less educated.

**Fig 2:**
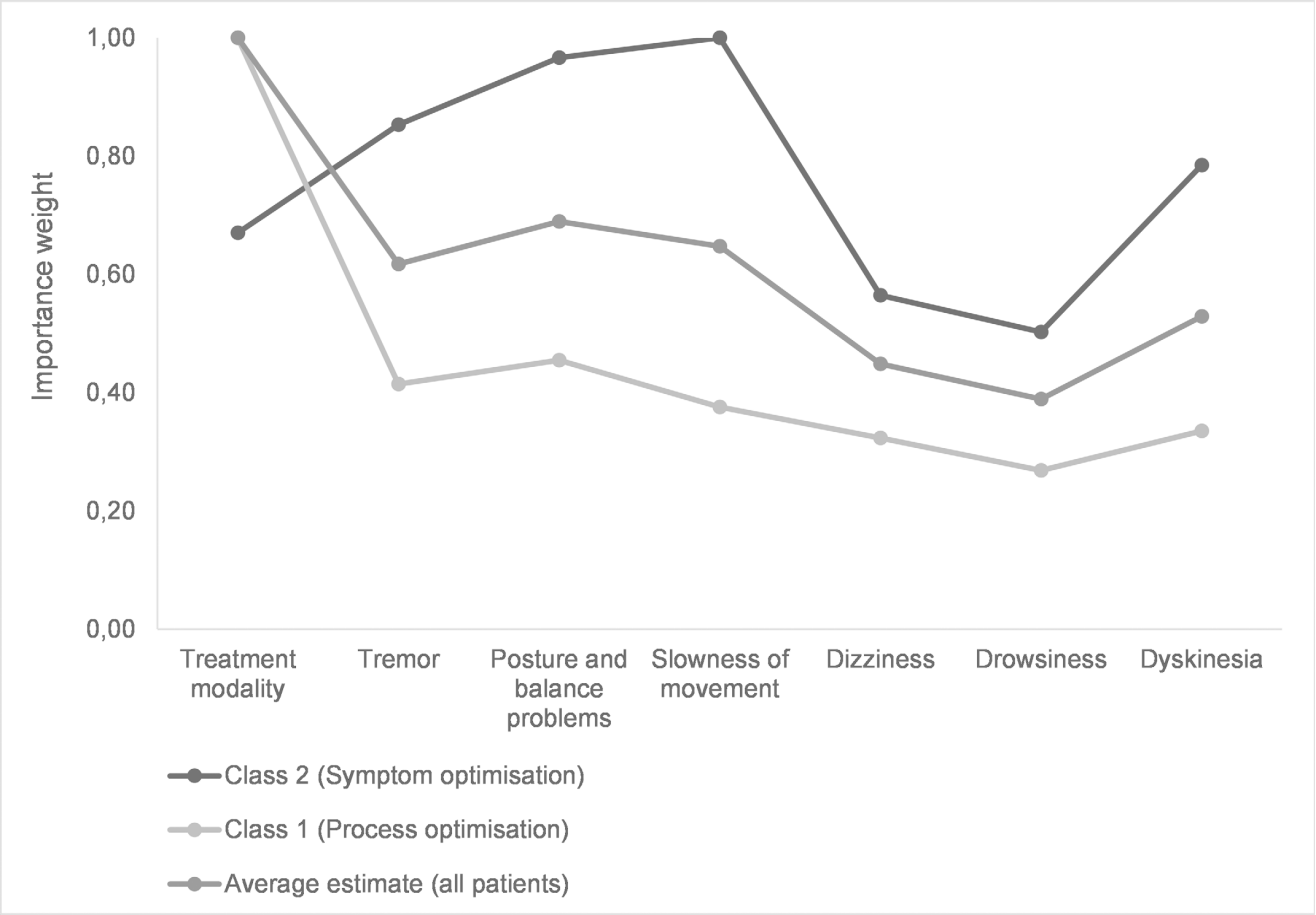
Importance weights of the attributes estimated from the conditional logit analysis and latent class analysis.

The conditional logit model included data for 192 patients and showed that patients perceived the option of neurosurgery as least desirable and ‘oral intake of medication’ as most desirable (Table 3, column 3). Fig 2 shows that treatment modality had the greatest impact on the perceived desirability of treatment (importance weight of 1). This was followed by two attributes related to treatment efficacy: the effect of treatment on reducing posture and balance problems (0.69) and slowness of movement (0.65). Of the side effects, suffering from dyskinesia was perceived as least desirable (0.53), followed by dizziness (0.45) and drowsiness (0.39). However, the occurrence of described side effects (dizziness, drowsiness and dyskinesia) had less impact on the perceived desirability of treatment than the treatment’s effect on motor symptoms. These findings are confirmed by the results of the direct assessment, where 67% of patients thought the perceived benefits of treatments were (very) influential in treatment choice while only 35% of patients considered the side effects of treatments to be (very) influential.

**Table 3:** Treatment desirability in Parkinson’s Disease based on conditional logit analysis and latent class analysis (N = 192).

Attribute	*Level*	Average Estimate^§^	Class 1: Patients preferring ‘Process optimisation’	Class 2: Patients preferring ‘Symptom optimisation’
Treatment modality	*Oral tablets*	1.83 (0.08)*	2.10 (0.11)*	1.20 (0.20)*
	*Pump*	-1.91 (0.08)*	-2.07 (0.11)*	-1.52 (0.17)*
	*Neurosurgery*	-2.74 (0.09)*	-4.36 (0.21)*	0.60 (0.24)*
Tremor	*Seldom to never*	1.66 (0.09)*	1.64 (0.11)*	1.77 (0.17)*
	*Sometimes*	0.21 (0.10)*	0.32 (0.12)*	0.08 (0.19)
	*Often*	-1.17 (0.09)*	-1.03 (0.14)*	-1.69 (0.17)*
Posture and balance problems	*Seldom to never*	1.70 (0.09)*	1.70 (0.11)*	1.82 (0.16)*
	*Sometimes*	-0.03 (0.10)	0.08 (0.12)	-0.24 (0.19)
	*Often*	-1.46 (0.09)*	-1.23 (0.12)*	-2.11 (0.17)*
Slowness of movement	*Seldom to never*	1.70 (0.09)*	1.59 (0.11)*	2.03 (0.17)*
	*Sometimes*	0.26 (0.10)*	0.44 (0.12)*	-0.15 (0.19)
	*Often*	-1.26 (0.09)*	-0.84 (0.13)*	-2.03 (0.17)*
Dizziness	*Seldom to never*	1.10 (0.09)*	1.11 (0.11)*	1.20 (0.18)*
	*Sometimes*	0.08 (0.10)	0.15 (0.12)	-0.07 (0.19)
	*Often*	-0.95 (0.10)*	-0.98 (0.13)*	-1.09 (0.18)*
Drowsiness	*Seldom to never*	1.16 (0.09)*	1.18 (0.12)*	1.21 (0.18)*
	*Sometimes*	0.23 (0.10)*	0.37 (0.12)*	-0.03 (0.19)
	*Often*	-0.62 (0.10)*	-0.55 (0.13)*	-0.83 (0.19)*
Dyskinesia	*Seldom to never*	1.36 (0.09)*	1.28 (0.11)*	1.66 (0.17)*
	*Sometimes*	-0.12 (0.10)	-0.02 (0.13)	-0.28 (0.19)
	*Often (ref)*^*§§*^	-1.06 (0.09)*	-0.89 (0.13)*	-1.53 (0.18)*
**Class probability model**^*§§§*^				
Constant			(ref)	0.56 (0.41)
Employment			-	0.48 (0.23)*
Experience with advanced treatments			-	1.13 (0.27)*
Disease duration			-	-0.07 (0.04)*
**Average class probability**			0.704	0.296
**Class membership**			N = 135	N = 57

^*§*^ A high positive part-worth utility reflects that the attribute-level is likely to be preferred relative to other attribute levels (the opposite is true for negative part-worth utilities).

^*§§*^ The data were entered using the effect-coding system. The part-worth utility of the reference category can be calculated as -1 * (the sum of the estimated part-worth utilities)

^*§§§*^ The membership predictors significantly improved the model fit compared to a model including no predictors (log likelihood -4626–4533, P < 0.001).

* p < 0.05

Columns 4–5 in Table 3 reveal the preference data of the two subgroups which were identified through latent class analysis. Both classes follow the previous observation that the benefits of treatment were more influential than the possible side effects. The importance weights of class 1 resemble the importance weights of the average estimate, except that it is even more important to optimise the treatment modality (Fig 2). The preference patterns of class 2 are very different from the average estimate and class 1, because the attributes related to motor symptoms had the greatest impact on treatment desirability. The focus of class 2 was on slowness of movement (importance weight of 1.0), posture and balance problems (0.97), and tremors (0.85). Furthermore, a notable difference was that these patients preferred neurosurgery over pump infusion while the average estimate and patients in class 1 preferred pump infusion over neurosurgery. Next, potential predictors were added to the model to determine whether certain patients are more likely to belong to class 2. In comparison with class 1, class 2 includes an above-average number of patients who had experienced advanced treatments (OR: 3.1) and who were still employed (OR: 1.3). In addition, a small significant difference was found between disease duration in both classes, with patients with a shorter disease duration more likely to belong to class 1 (OR: 0.19). Based on the highest probability to belong to a class, 57 patients were assigned to class 1 and 135 patients were assigned to class 2. Prevalence of significant predictors were then: employment (27.2% in class 1 versus 13.1% in class 2), experience with advanced treatment (32.7% versus 9.56%), and disease duration (6.8 years versus 7.3 years). We found no evidence that age, gender, education, independence, or quality of life scores significantly influenced the preference patterns in the calculated latent class model.

### Patient involvement

The roles patients preferred in decision-making about treatment were 8% active, 37% active-shared, 30% collaborative, 24% passive-shared and 1% passive (N = 212) (Table 4). The roles that patients reported actually experiencing were 12% active, 59% collaborative, and 29% passive. Patients’ preferred and perceived roles were significantly associated (x^2^ (8) = 40.1, P < .001). For 78% of patients it can be concluded that they approximately experienced their preferred role during decision-making. In total, relatively few patients (11.3%) experienced extreme discordance between their preferred role and their perceived role (Table 4). The majority of patients (45%) preferred to take the lead in treatment decisions, however 10.8% preferred a more passive or collaborative decision role. In addition, the direct questions revealed that only 10% of patients take into account the advice of family or friends when deciding about treatment, and assistance needed from partner was only relevant for 23% of patients.

**Table 4:** Congruence between the patient’s preferred and perceived decision role in treatment decision making (N = 212).

*Perceived Decision Role → Preferred Decision Role ↓*	Active: Treatment was largely chosen by myself	Collaborative: Treatment was chosen in collaboration with the doctor	Passive: Treatment was largely chosen by the doctor	Total
**Active**: I prefer to make the final treatment decision.	5*	9**	3**	17 (8%)
**Active-shared**: I prefer to make the final treatment decision after seriously considering my doctor’s opinion.	17*	51*	11**	79 (37%)
**Collaborative**: I prefer that my doctor and I share responsibility for deciding which treatment is best.	3	41*	20	64 (30%)
**Passive-shared**: I prefer that my doctor makes the final treatment decision, but seriously considers my opinion.	1**	23*	26*	50 (24%)
**Passive**: I prefer to leave all treatment decisions to my doctor.	-	-	2*	2 (1%)
**Total**	26 (12%)	124 (59%)	62 (29%)	212 (100%)

x^2^ (8) = 40.1, P < .001

* Patients who approximately had their preferred role during decision making.

** Patients who experienced extreme discordance in their preferred and perceived decision role during decision making

## Discussion

The objective of this study was to elicit patient preferences about motor symptoms, side effects, and process characteristics of the main treatments in PD and to measure patients’ preferred and perceived involvement in decision-making about treatment. The best-worst scaling data indicated that the occurrence of described side effects (dizziness, drowsiness, and dyskinesia) has less impact on the perceived desirability of treatment than the treatment’s effect on motor symptoms. In contrast, a study of Hattori et al. in Japan (N = 371) found that more than half of the study participants preferred to avoid side effects (dyskinesia, drowsiness, constipation etc.) rather than obtain effective relief from bradykinesia [28]. However, this number gradually decreased with increasing symptom severity. The difference between studies might be explained by cultural differences or by the way the questions were posed (direct/indirect). In our study dyskinesia was identified as the most influential side effect for treatment preference; however compared to dizziness and drowsiness, dyskinesia was the side effect from which the fewest patients suffered. This might be explained by findings in other studies that patients who had not yet experienced dyskinesia were more concerned about avoiding dyskinesia than patients who had already developed dyskinesia [28, 29].

Furthermore, two preference patterns were found in the patient sample. One class focused largely on optimising the process of care, while the other class focused more on controlling motor symptoms. Patients who had experienced advanced treatments, who were still employed, or who had a shorter disease duration were more likely to belong to the latter class. One can only speculate about the reasons why this was the case. It may be that experience with advanced treatment increases its perceived value (or reduces the disutility of its negative aspects) and patients are less interested in process optimisation. On the other hand, these patients may have had different preferences than the average patient before choosing advanced interventions [30]. Second, Murphy et al. (2013) found that employed patients reported slowness and tremor as their greatest occupational challenges and therefore these motor symptoms might be perceived to be more important by this class in our study [31]. Although disease duration significantly differs between the two preferences groups one it is arguable whether this difference is clinically relevant (6.8 years versus 7.3 years). However, for patients with a recent onset (short disease duration), it may be assumed that treatment still has a direct, apparent effect on motor symptoms and thus they may be perceived as more important. We did not find evidence that age influenced preference patterns in our model. However, it is unlikely that the decision-making process is the same in a 45-year-old man as in an 80-year-old man, though we did not find evidence for this in the current model.

Previous studies have indicated that it is important to provide care that is tailored to each PD patient’s individual values and preferences [14, 15]. Our study revealed subgroups with varying preferences, indicating that there is no “one-size-fits-all” treatment and that treatment decisions have to be individualised. Fortunately, we found that many patients (45%) preferred an active role in the treatment decision-making process. Only 1% of patients prefer to leave the treatment decision entirely up to the doctor, while 24% of patients reported that in their case the treatment decisions were largely made by their doctor. If patients desire more involvement in the decision-making process, it is essential for the doctor to know what is important to patients. We are currently studying the possibility of developing a value clarification tool to facilitate individual treatment decisions for advanced treatments in PD. This tool will help physicians to propose individualised treatment strategies which are tailored to the patient’s preferences and enhance collaborative or shared decision-making. Understanding the advantages and disadvantages of the available treatment options will help patients feel more involved and more certain about the relevance and likely efficacy of their treatment and may therefore enhance adherence to treatment [17].

### Limitations

Although the attribute selection process in this study can be criticised because we only interviewed a limited number of patients through convenience sampling (selection bias), we did rely on a process of open, semi-structured and structured interview questions. We feel that one can attach value to all answers, as the answers patients gave spontaneously highlighted their main daily concerns (which might not be related to specific drugs), while providing a detailed list of symptoms and side effects might have brought other concerns to the surface. With a different group of patients, the definite selection of attributes might have been altered. However, the background characteristics of this sample and the final patient sample were comparable and we do feel that this article highlights important trade-offs between attributes that patients make in expressing preferences for treatment. However, this does not mean that our attribute selection process was comprehensive: the results of our study only hold true for the importance of the symptoms and side effects that were part of the treatment scenario. Lastly, the physical aspects of PD are the defining characteristics of the disease and, understandably, patients focused on those during the interviews. Our final selection of attributes did not include non-motor symptoms. However, literature suggests that non-motor symptoms have, as a whole, a greater impact on quality of life than motor symptoms [32, 33]. Further research should focus on the relative value of non-motor symptoms versus motor symptoms.

In this study, the method best-worst scaling was selected to elicit preferences. Compared to other trade-off methods such as discrete choice experiments, time trade-off, and standard gamble, literature suggests that best-worst scaling has a lower cognitive burden [18, 34, 35]. Rating scales would probably have been easier for the patient to complete, but then no statements could have been made about the relative desirability of treatment attributes. However, overall 45 of the 132 submitted paper-questionnaires had to be removed from the sample (34%). Older (>75) and less educated patients were excluded more often, which might be explained by the initial and declining cognitive abilities as a result of age, or their disease [36]. Completion of the questionnaire together with a researcher or during an interview would probably have improved the reliability of the answers, but would have introduced the risk of interviewer bias and, in any case, was not possible due to time and budget constraints.

For the data collection, there was more uncertainty regarding the non-hospital based sample, e.g. it was explicitly stated that patients with atypical parkinsonism should not fill out the questionnaire, but we cannot be sure this did not happen. In the analysis phase, the hospital and non-hospital sample were merged. Although in the latter sample the patients were younger and more frequently employed, the two samples were almost equally divided among the two estimated latent classes (48–52%).

For the Control Preference Scale questions, the different operationalisation of the perceived and preferred decision roles led to difficulties with estimating the level of (dis)agreement between them. Therefore we only estimated extreme discordance between the roles and approximated the level of agreement. However, this might have led to a bias in the results found. Had the same number of answer categories been used, it would have been possible to calculate the level of (dis)agreement using Cohen’s kappa. In addition, the Control Preferences Scale does not recognise the decision role of the partner or caregiver, while in (later stages of) PD this role becomes very important [37]. In our study, 20% of the patients completed the questionnaire together with their partner or caregiver, which also shows their interest in the subject.

## Conclusions

Our study revealed differences in the way subgroups of patients weigh the different attributes of treatment, indicating there is no “one-size-fits-all” treatment possible and decision-making about treatment needs to be individualised. It was found that patients who had experienced advanced treatments, who were still employed, or who had a shorter disease duration were more likely to focus their treatment on controlling motor symptoms rather than on optimising the process of care. In addition, our study has shown that many patients prefer an active role in decision-making about treatment. Both results stress the need for physicians to know what is important to patients and to share treatment decisions to ensure that patients receive the treatment that aligns with their preferences.

## Supporting Information

S1 Appendix Selection of attributes and levels. (DOCX)
