# Supplementary material for: Involving Patients in Weighting Benefits and Harms of Treatment in Parkinson's Disease
Source: PLoS One. 2016 Aug 30;11(8):e0160771. doi: 10.1371/journal.pone.0160771 (PMC5004993; doi:10.1371/journal.pone.0160771)
Supplement: S1 Appendix — (DOCX) [file pone.0160771.s001.docx]

**S1 Appendix: Selection of attributes and levels.**

Introduction

In a preliminary study, the identification of relevant symptoms, side effects and process characteristics in Parkinson’s Disease (PD) took place through qualitative interviews with 18 patients. This provided the basis for identifying the full set of attributes that characterise PD treatments and influence patients’ Health Related Quality of Life (HRQoL). Subsequently, these results were discussed in a project meeting and based on the qualitative and quantitative importance of these characteristics in patients’ daily life attributes were selected for the preference experiment.

Methods

The interviews with patients were semi-structured using a framework of important themes influencing HRQoL in PD (table 1). Patients were first asked to describe their health using the Parkinson-Disease Questionnaire-39 (PDQ-39) [1] and the EuroQol-5D-5L (EQ5D-5L) [2]. With this information the interviewer got an overall impression of the health status of the patients and used this information to structure the interviews and to decide which health domains were important to discuss (semi-structured interviews). Hereafter, patients were asked about the main characteristics of their treatment, the positive and negative outcomes, the influence on HRQoL and other themes listed in table 1. The interviews were tape recorded and the expected duration of the interviews was about an hour.

**Table 1. Structured themes during the interviews.**

| **Global themes** | **Points of discussion** |
| --- | --- |
| Personal characteristics | Age, marital status, children, employment |
| Global health status | Diagnosis, duration, medication use, health problems, PDQ-39 and EQ5D-5L assessment. |
| Effect of symptoms on HRQoL | Efficacy, symptom suppressing (duration) |
| Effect of side-effects or motor-fluctuations on HRQoL | Side effects (frequency, duration), influence of daily life, experience with dyskinesia, wearing off, on-off effects and/or freezing |
| Impact of treatment (process) characteristics on HRQoL | Treatment regime, experience, difficulties, satisfaction, quitting/adhering to a drug, advanced treatment (brain surgery, pump) |

*Data analysis*

For the PDQ-39 dimension scores were obtained and a summary index was calculated. For the EQ5D-5L an overall index utility value was retrieved. The interviews were transcribed verbatim and coded and analysed in ATLAS.ti (software for the qualitative analysis of large bodies of textual and audio data) [3]. The final result was a table ranking the frequency of symptoms and side effects (attributes) and their impact on HRQoL according to these patients. Discussion with a project team consisting of a neurologist, a hospital pharmacist, a rehabilitation specialist, three health sciences researchers, and two patients was used to narrow the list of attributes. The project team selected attributes based on: the qualitative and quantitative importance of these attributes of treatment in the daily life of patients (interview results), and the limitations of the chosen stated preference method, which only allowed for inclusion of between four and eight attributes. More-over, we aimed to reach attribute balance (i.e. include equal positive and negative outcomes of treatment choice and to make sure that the selected attributes were typical symptoms and side effects of PD and not too general.). At the end of the project meeting the goal was to have selected attributes that highlights important trade-offs between attributes that patients make in expressing preference for treatment.

**Interview results**

Table 2 shows the sociodemographic and clinical characteristics of the 18 patients enrolled in the interviews. Table 3 shows the results of the PDQ-39: patients suffered the most from problems in the domains of ‘activities of daily living and mobility’. Only few problems were identified in the stigma and social support domains.

**Table 2. Socio-demographics and clinical characteristics (N=18).**

| **Sample characteristics (N=255)** | **N (%) *or* mean** ± **SD (min, max)** |
| --- | --- |
| **Gender**  Man  Woman | 13 (72.2)  5 (11.1) |
| **Age (years)** | 66 ± 7.0 (54, 79) |
| **Employment status**  Employed  Disabled / unable to work  Retired | 3 (16.7)  4 (22.2)  11 (61.1) |
| **Marital state**  Single  With partner (no children living at home)  With partner and children living at home | 2 (11.1)  15 (83.3)  1 (5.6) |
| **Disease duration** | 8.0 ± 7.15 (1, 22) |
| **Current treatment**^¥^  Oral medication  Continuous pump infusion (subcutaneous)  Continuous pump infusion (intraduodenal)  Neurosurgery | 18 (100.0)  -  1 (5.6)  3 (16.6) |
| **Current oral medication treatment**  Levodopa  Dopamine agonist  Combination therapy | 7 (38.9)  2 (11.1)  9 (50.0) |

**Table 3. Health-related quality of life questionnaires (N=18).**

| Variables | Mean (standard deviation) |  |  |
| --- | --- | --- | --- |
| **PDQ-39**  Mobility | 32.78 (25.67) |  |  |
| Activities of daily living | 38.89 (23.31) |  |  |
| Emotional well-being | 23.61 (17.21) |  |  |
| Stigma | 9.03 (13.08) |  |  |
| Social support | 12.96 (18.35) |  |  |
| Cognitive impairment | 37.5 (15.46) |  |  |
| Communication | 30.06 (15.19) |  |  |
| Bodily discomfort | 30.39 (15.54) |  |  |
| **PDQ-39 Summary Index** | 30.38 (15.5) |  |  |
| **EQ5D-5L Index Value** | 0.69 (0.25) |  |  |
| **EQ5D-VAS** | 68.72 (11.47) |  |  |

**Table 4. Symptoms and side effects and their influence on HRQoL(N=18).**

|  | no. of patients suffering | Influence on HRQoL (according to most patients) |
| --- | --- | --- |
| **Symptoms** |  |  |
| Postural instability | 15 | Major |
| Tremors | 13 | Major |
| Slowness of movement (reduced gait, leg dragging, reduced arm swing) | 10 | Major |
| Freezing | 8 | Major |
| **Rigidity, muscle stiffness** | 7 | Major |
| Anxiety – Depression | 3 | Major |
| Constipation | 2 | Minor |
| Writing | 2 | Minor |
| Loss of smell | 1 | Minor |
| Crying | 1 | Moderate |
| Swallowing | 1 | Moderate |
| Drooling | 1 | Moderate |
| **Side effects** |  |  |
| Dyskinesia | 10 | Major |
| Drowsiness (sleepy, daytime sleepiness) | 8 | Major |
| Dizziness (orthostatic hypotension) | 8 | Moderate |
| Wearing off | 8 | Moderate |
| Constipation, diarrhea and incontinence | 7 | Moderate |
| On-off response | 5 | Major |
| Nausea, stomach pain and or vomiting | 5 | Minor |
| Falling asleep at night | 4 | Moderate |
| Headache | 3 | Moderate |
| Confusion, hallucinations and paranoia | 3 | Major |
| Waking up too early | 2 | Minor |
| More frequent dreams and nightmares | 1 | Moderate |
| Dislocation intestinal tube | 1 | Minor |

Table 4 shows the final result of the interviews: a table ranking the frequency of symptoms and side effects (attributes) and their impact on HRQoL according to most patients. Since not all patients had experience with advanced treatment, this theme was not quantified. However, the qualitative results will be reported.

Impact of treatment (process) characteristics on HRQoL

The last theme of the interview was how the process characteristics of different treatments in PD affect patient’s daily life and HRQoL. All patients took oral medication and thought this only had a minor influence on their HRQoL. There were only four patients who had experienced brain surgery or were currently wearing a pump. At first instance, these patients had doubts about starting advanced treatments, but in the end they adapted to their situation because it brought them relief of symptoms. The fourteen non-experienced patients were asked to think of undergoing brain surgery or using a pump (and likewise to consider all routines, operations and factors involved). All patients were very firm in their opinion and were certain that starting with advanced treatments would have a major influence on their daily life and HRQoL, despite the benefit of expected symptom reduction. With respect to brain surgery, most patients were aware of the risk of complications, but the prospect of being awake during deep brain stimulation provoked most anxiety and fear. Besides the surgery itself, patients expected the use of the neurostimulator to be complicated and difficult, as well as with a pump. With a pump patients stood negatively against carrying the pump at all times, changing the drug cassette, rinsing the intestinal tube or changing the infusion needle/line. All in all, the advanced treatment modalities will have a major influence on HRQoL.

**Project meeting**

After deliberation, one process attribute (treatment modality), three motor symptoms (tremor, slowness of movement, and posture and balance problems) and three side effects (dizziness, drowsiness, dyskinesia) were selected for inclusion in the preference task.

The process attribute

The selection of the process attribute reflected interviewed patients expressing concerns regarding the impact of surgery and pump procedures on their daily life, despite the expected reduction in symptoms.This highlights an important trade-off in expressing preference for treatment. Therefore the process attribute was operationalized as treatment modality: whether the treatment required brain surgery, continuous infusion of medication via a pump, or oral medication.

Motoric symptoms:

The physical aspects of PD (motoric symptoms) are the defining characteristics of the disease and, understandably, patient’s focus most on those during the interviews. The three selected symptoms for the preference instrument were: tremor, posture/balance problems, and slowness of movement. These symptoms occurred most, are easily recognized and had a major impact on HRQoL according to interviewed patients. In the project team the exclusion of rigidity / muscle stiffness was heavily debated, but eventually the project team selected the attributes which were most often reported by the patients in the interviews.

Side effects:

To keep a balance between symptoms and side effects, also three side effects were selected: dyskinesia, drowsiness, and dizziness. We aimed to select side effects which were recognizable to most patients, and side-effects which were typical to PD and not too general. The interview results showed that dyskinesia occurred the most often and had the largest impact on patient’s daily life, because of the duration, the unpredictable character, and the obstruction of daily tasks. Secondly, side effects were most often reported in the sleeping domain and the selected attribute drowsiness was defined as extensive daytime sleepiness. Nausea, stomach pain, vomiting, diarrhea, constipation also frequently occurred, but most had only a limited to moderate impact on HRQoL. Besides, nausea and stomach pain are examples of side effects which are common in taking oral medication. Lastly, dizziness (lightheadedness caused by orthostatic hypotension) was selected at the cost of ‘hallucinations and paranoia’. Although the latter had a greater impact on HRQoL, it was only reported by three patients.

Level assignment

The next step was to describe the variation in possible outcomes for each attribute. In order to reduce the cognitive difficulty, three qualitative levels were chosen to represent the burden of symptoms and side effects in the treatment scenarios (seldom to never, sometimes, and often suffer from). The attribute treatment modality was described as the oral intake of tablets, continuous pump infusion of medication, and neurosurgery.Table 5 displays the final selection of attributes, their descriptions and levels, which served as the input for the preference experiment.

**Table 5. Selected attributes and levels (descriptions)**

| **Attributes** | **Description** | **Level operationalization** |
| --- | --- | --- |
| Treatment modality | The path by which a  treatment enters (or is  applied to) the body. | Oral tablets  Medication through pump infusion  Brain surgery |
| Tremor | Rhythmic  muscle contraction | Seldom to never suffer from  Sometimes suffer from  Often suffer from |
| Posture and balance problems | Abnormal axial postures and loss of postural  reflexes. | Seldom to never suffer from  Sometimes suffer from  Often suffer from |
| Slowness of  movement | Difficulties with planning, initiating and executing  movement | Seldom to never suffer from  Sometimes suffer from  Often suffer from |
| Drowsiness | Excessive daytime sleepiness | Seldom to never suffer from  Sometimes suffer from  Often suffer from |
| Dizziness | Lightheadedness, effect of  orthostatic hypotension | Seldom to never suffer from  Sometimes suffer from  Often suffer from |
| Dyskinesia | Repetitive, involuntary  muscle movement | Seldom to never suffer from  Sometimes suffer from  Often suffer from |

References

1. Jenkinson C, Fitzpatrick R, Peto V, Greenhall R, Hyman N. The Parkinson's Disease Questionnaire (PDQ-39): development and validation of a Parkinson's disease summary index score. Age Ageing. 1997;26(5):353-7.

2. Herdman M, Gudex C, Lloyd A, Janssen M, Kind P, Parkin D, et al. Development and preliminary testing of the new five-level version of EQ-5D (EQ-5D-5L). Qual Life Res. 2011;20(10):1727-36.

3. ATLAS.ti Scientific Software Development Company. ATLAS.ti. Berlin, Germany: GmbH.
